# A gangliocytic patially glandular paraganglioma with lymph node metastasis

**DOI:** 10.1186/1746-1596-9-63

**Published:** 2014-03-20

**Authors:** Huijuan Shi, Ju Han, Ni Liu, Ziyin Ye, Zhixun Li, Zhi Li, Tingsheng Peng

**Affiliations:** 1 Department of Pathology, the First Affiliated Hospital of Sun Yat-sen University, 58, Zhongshan Road II, Guangzhou 510080, P. R. China

**Keywords:** Gangliocytic paraganglioma, Glandular component, Lymph node metastasis, Duodenum

## Abstract

Gangliocytic paraganglioma (GP) is an infrequent neuroendocrine tumor usually with three elements as epithelioid cells, spindle-shaped cells and ganglion-like cells, which is generally regarded as a benign tumor. Only a few cases with lymph node metastasis have been reported. Herein, we reported a 47-year-old man of GP with distinct glandular component embedded in the spindle tumor cells in the primary tumor and the metastatic lymph nodes. The immunohistochemical profile was helpful to give the final diagnosis as gangliocytic paraganglioma. Here, we added one more GP case with regional lymph nodes metastasis. And particularly, there were small amount of distinct glandular component both in the primary tumor and the metastatic lymph nodes, which indicated that adenocarcinoma might coexist with GP. And GP should also be distinguished from carcinoid tumor, paraganglioma, ganglioneuroma, or GIST.

## Letter to the editor

Gangliocytic paraganglioma (GP) is an infrequent neuroendocrine tumor, usually being found in the second portion of the duodenum. The histological diagnosis requires the identification of three elements as epithelioid cells, spindle-shaped cells and ganglion-like cells [1,2]. The tumor cells arrange in solid and trabecular pattern, mainly comprising spindle cells, mixed with nests of epithelioid cells and large cells with gangliocytic differentiation. Few cases were reported to contain distinct epithelial component forming glandular structure. Generally, this tumor is regarded as a benign tumor, but a few cases with lymph node metastasis have been reported before (Table 1). In addition to the rarity of the tumor, the present case suggests the malignant potency of this tumor. Herein we reported a rare case of gangliocytic paraganglioma with lymph node metastasis, comprising distinct glandular component in primary tumor and the metastatic lymph nodes.

**Table 1:** Gangliocytic paraganglioma cases with lymph node metastasis

**Reference**	**Published year**	**Age (years)**	**Sex**	**Chief clinical presentation**	**Size (mm)**
Büchler et al. [7]	1985	50	Male	Gastrointestinal bleeding	30
Inai et al. [8]	1989	17	Male	Hematoemesis	20
Hashimoto et al. [9]	1992	47	Male	Incidental findings	65
Dookhan et al. [10]	1993	41	Male	Abdominal pain	25
Sundararajan et al. [11]	2003	67	Female	Incidental findings	50
Bucher et al. [12]	2004	31	Female	Anemia, subclinical	30
Wong et al. [13]	2005	49	Female	Melena	14
Witkiewicz et al. [14]	2007	38	Female	Abdominal pain	15
Mann et al. [15]	2009	17	Female	Abdominal pain, vomiting, weight loss	NR
Okubo et al. [16]	2010	61	Male	Epigastralgia, tarry stool	30
Saito et al. [17]	2010	28	Male	Gastrointestinal bleeding, anemia	17
Uchida et al. [18]	2010	67	Female	Anemia	NR
Ogata et al. [19]	2011	16	Male	Gastrointestinal bleeding, anemia	35
Barret et al. [20]	2012	51	Female	Anemia	35

NR: not reported.

**Figure 1:**
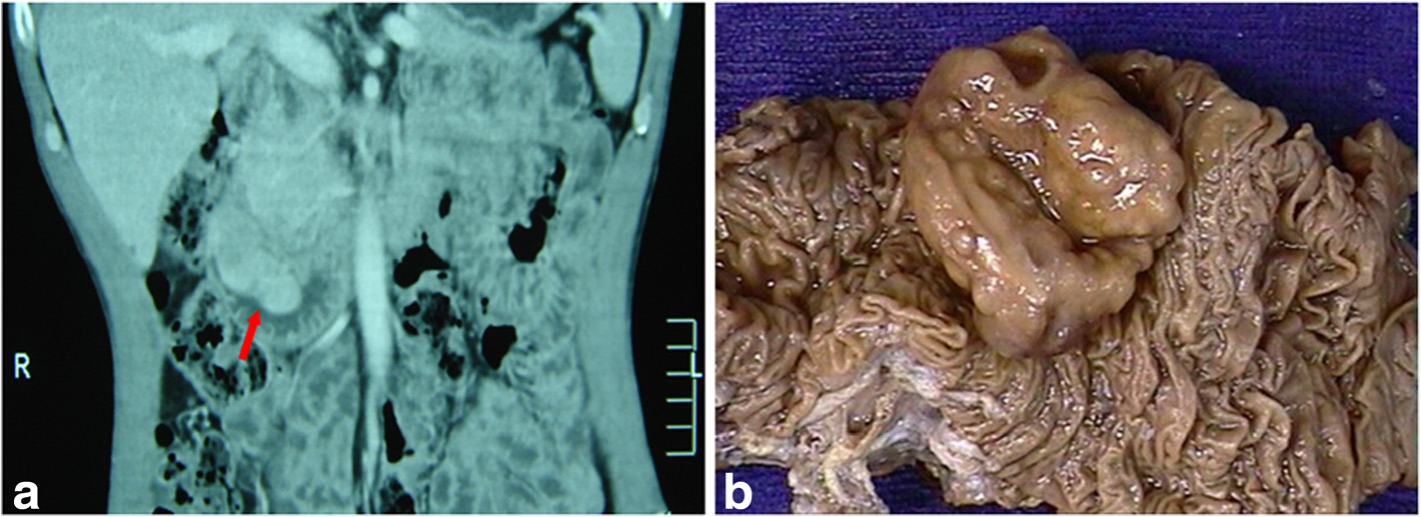
Computed tomography (a) and surgical specimen (b) revealed a tumor at the papilla of Vater in duodenum.

The patient present here, a 47-year-old man with an unremarkable previous medical history, had a 4-month history of left lower quadrant abdominal pain before admission. He had experienced weight loss of approximately 5 kg during the previous 4 months and had been previously treated with H_2_ blockers and proton pump inhibitors without significant relief. CT scan of the abdomen showed a neoplasm with 6.6 × 4.0 × 3.5 cm at the papilla of Vater in duodenum (Figure 1a). Peripancreatic lymph nodes swelled to the largest diameter as 3.0 cm. No dilatation of the biliary or pancreatic duct was observed. A gastrointestinal endoscopy detected a 4.0 × 2.5 cm, polypoid, ulcerated ampullary tumor in duodenum. Endoscopic ultrasonography suggested that the tumor involved in the whole duodenal mucous layer and the lymph node around enlarged. Pancreatcoduodenectomy accompanied by peripancreatic lymph node dissection were performed. Intraoperative biopsy of the enlarged lymph nodes showed regional atypical glandular component in the lymph nodes, leading to the misimpression as a metastatic neuroendocrine carcinoma firstly.

**Figure 2:**
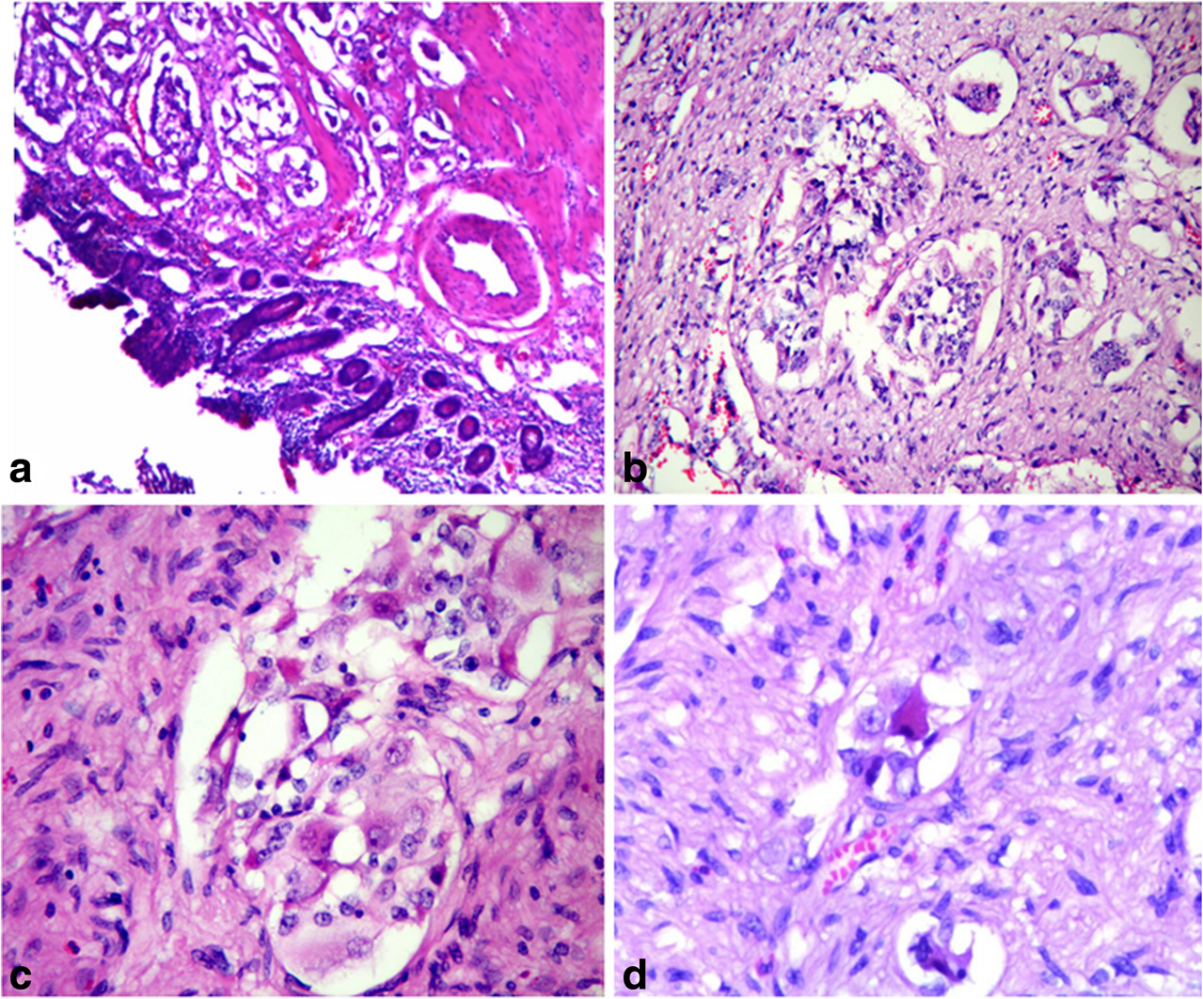
**Submucosal location of the tumor in the periampullary region (a, H&E 100×).** The tumor consists of epithelioid cells forming nests (**b**, H&E 200×), fascicles of spindle cells and ganglion-like cells (**c**, **d**, H&E 400×).

**Figure 3:**
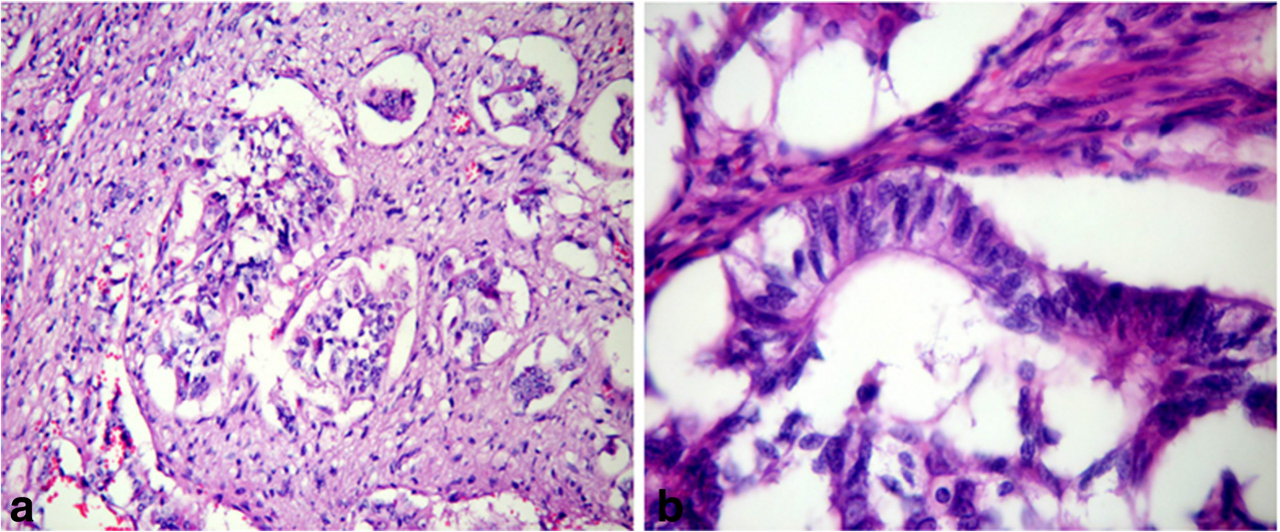
Focal glandular structures in the primary tumor (a, H&E 200×, b, H&E 400×).

**Figure 4:**
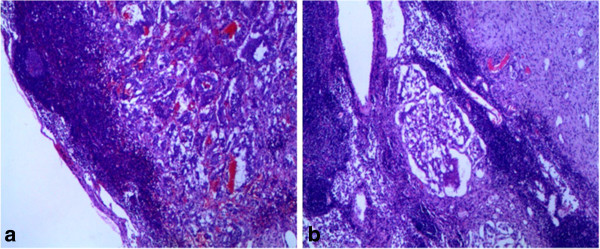
Metastatic tumor in a lymph node composed of epithelial cells, spindle cells and ganglion cells (a, H&E 100×, b, H&E 200×).

**Figure 5:**
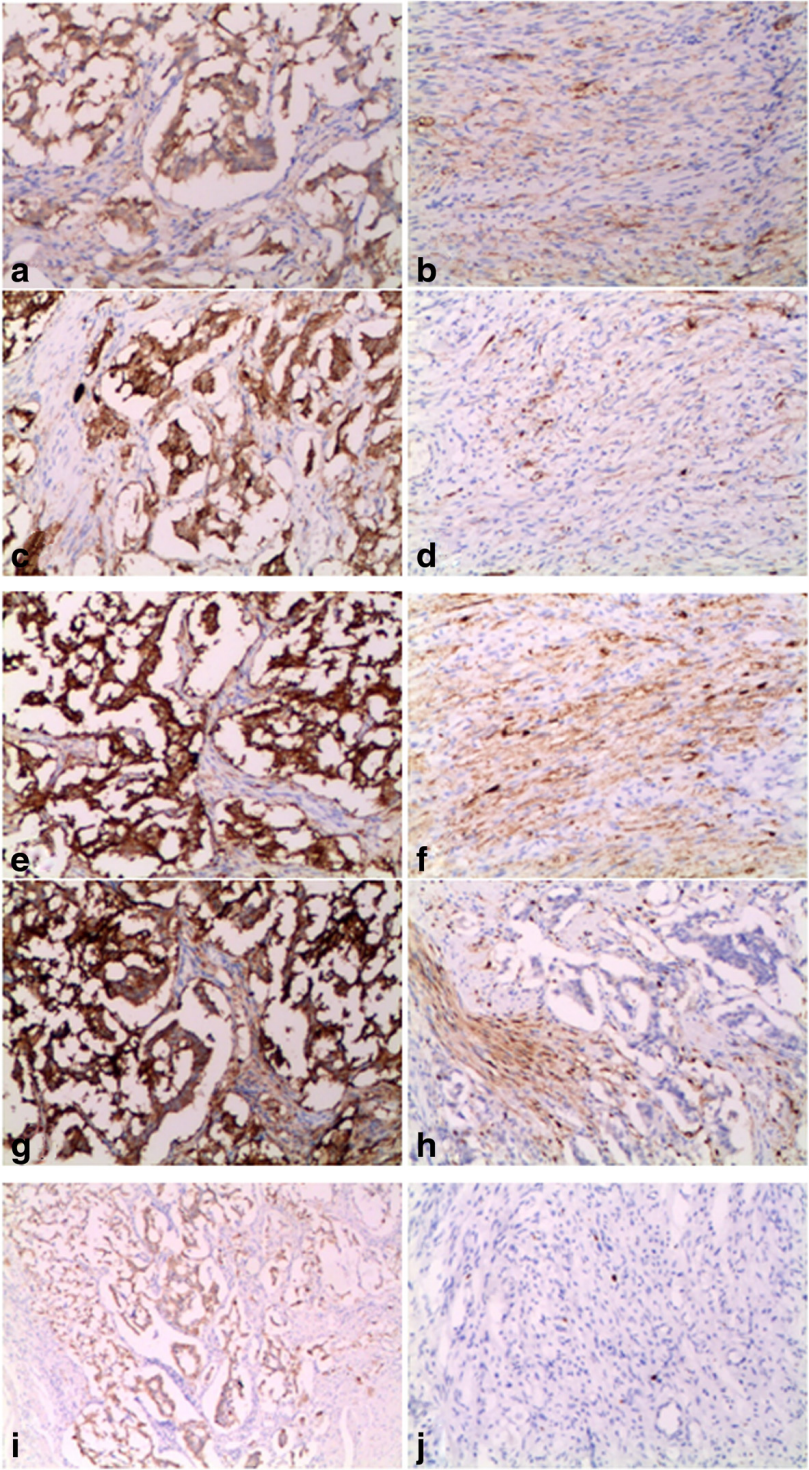
**Immunohistochemistry staining of the tumor tissue.** Epithelioid cell nests are positive for NSE **(a)**, CgA **(c)**, Syn **(e)**, CD56 **(g)**, CK **(i)**. Spindle cells are positive for NSE **(b)**, CgA **(d)**, Syn **(f)**, S-100 **(h)**. Ki-67 staining shows the proliferative rate is less than 1% **(j)**.

Gross examination revealed the surgical specimen comprising a portion of duodenum with ampulla, the gallbladder, and head of the pancreas. A 4.0 × 4.0 × 2.3 cm polypoid tumor was found at the papilla of Vater (Figure 1b). A total of twenty lymph nodes were also removed respectively, including 7 peripancreatic lymph nodes with greatest diameter as 3.0 cm, 5 suprapyloric and 8 subpyloric lymph nodes. Microscopically, the tumor were localized in submucosal layer, invading a part of the muscularis propria (Figure 2a), being mainly consisted of spindle cells, with some nests of epithelial cells (Figure 2b-c), and scattered big ganglion-like cells (Figure 2d). The spindle cells formed slender fascicles, with elongated and plump nucleus, and attenuated eosinophilic cytoplasm. The epithelial cells arranged in the nests and trabeculae, with round to oval-shaped nucleus and pale eosinophilic cytoplasm. The ganglion-like cells were rarely seen, with round nucleus, large conspicuous nucleolus, and abundant eosinophilic cytoplasm (Figure 2d). Specially, small amounts of distinct atypical glandular components were presented (Figure 3a-b). The tumor also invaded a portion of pancreatic tissue. Eight of the twelve lymph nodes were involved in metastatic tumor (Figure 4a-b). The atypical metastatic glandular components in the lymph nodes had caused the misimpression as metastatic neuroendocrine carcinoma on the initial frozen slides. Immunohistochemically, the neoplastic epithelial cells were positive for the epithelial and neuroendocrine marker as CK, Neuron-specific enolase (NSE), Chromogranin A (CgA), Synaptophysin (Syn), CD56. The spindle tumor cells arrouding the epithelial nests were positive for S-100, partly for NSE, CgA, Syn, CD56 and CD34, but negative for CK. The ganglion cells were characteristic positive for S-100. Ki-67 labeling index estimated less than 1%. CD117, Actin and Desmin were negative in all of the three components (Figure 5a-h). Based on all these clinicopathological features, we finally made a diagnosis of gangliocytic paraganglioma with glandular component and lymph nodes metastasis. To date, approximately two years routine follow-up after the surgery is established, and the patient remains well and no recurrence has been recognized. Because of the metastasis, the malignant potential of this tumor could not be excluded. A long time following up is needed to know exactly the prognosis.

GP is an infrequent neuroendocrine tumor usually appearing in the second part region of duodenum. The most common clinical manifestation is gastrointestinal bleeding (45.1%) due to mucosal erosion or ulceration, followed by abdominal pain (42.8%) and anemia (14.5%) [3]. GP has been known well after it was firstly reported by Dahl et al. and named entity by Kepes et al. [1,2]. Confirmation of three identical components comprising epithelial cells, spindle cells, and ganglion cells was essential for the diagnosis. GP should be distinguished from carcinoid tumor, ganglioneuroma, pigmented paraganglioma, and spindle cell tumors as GIST [4-6]. Immunohistochemical examination was also an important diagnostic clue to identify the three cellular components of GP.

In this case the epithelial component in the metastatic lymph nodes led to the thought as metastatic neuroendocrine carcinoma in frozen slides. With more than ten tissue blocks section, three components as spindle cells, epithelial cells, and ganglion cells through light on the diagnosis as gangliocytic paraganglioma. Immunohistochemical staining had confirmed the diagnosis. In this case, MIB-1 was estimated as less than 1%, suggesting the low proliferative rate of this tumor, which might not reflect the prognostic value in GP.

Although GP is generally considered as a benign periampullary lesion, however, it is very unwise to assume that this tumor must be a benign entity. Metastasis to regional lymph nodes by this tumor and/or local recurrence has been reported several times in the literature [7-20] (Table 1). Here, we added a GP case with lymph node metastasis to that list. Although there is still no distinct evidence that the lymph node metastasis indicating malignant prognosis, lymphovascular invasion may be a major factor in the malignant potential of GP. In hence, it was important to image the examination to investigate the possibility of lymph node metastasis before an operation.

Anders [21] had reported a GP case with an advanced duodenal adenocarcinoma coexisted. In our case we also found small amount of distinct glandular components besides three typical tumor cells of GP. Hence, it could not be excluded for the potential that adenocarcinoma coexist with GP at the same location. Although the patient remains well and no recurrence after nearly two years routine follow-up, a long time follow-up is needed to know whether there is a malignant capacity of this case.

Herein we presented a rarely gangliocytic patially glandular paraganglioma with lymph node metastasis. In addition to the rarity of the tumor, we wish to emphasize the pleomorphic morphologic features mimicking adenocarcinoma and the malignant potency of gangliocytic paraganglioma with lymph nodes metastasis.

## Consent

Written informed consent was obtained from the patient for publication of this Case Report and any accompanying images. A copy of the written consent is available for review by the Editor-in-Chief of this journal.

## Competing interests

The authors declare that they have no competing interests.

## Authors’ contributions

HS drafted the manuscript and performed the literature review. JH conducted the pathological examination and literature review. NL conducted the immunohistochemical staning. ZY participated in the final diagnosis. ZXL carried out the pathological examination. ZL participated in the immunohistochemical analysis. TP gave and reviewed the final histopathological diagnosis, and revised and gave final approval of the version to be published. The final manuscript was read and approved by all authors.
